# How important is EMT for cancer metastasis?

**DOI:** 10.1371/journal.pbio.3002487

**Published:** 2024-02-07

**Authors:** Toni Celià-Terrassa, Yibin Kang

**Affiliations:** 1 Cancer Research Program, Hospital del Mar Research Institute, Barcelona, Spain; 2 Department of Molecular Biology, Princeton University, Princeton, New Jersey, United States of America; 3 Ludwig Institute for Cancer Research Princeton Branch, Princeton, New Jersey, United States of America

## Abstract

Epithelial-to-mesenchymal transition (EMT), a biological phenomenon of cellular plasticity initially reported in embryonic development, has been increasingly recognized for its importance in cancer progression and metastasis. Despite tremendous progress being made in the past 2 decades in our understanding of the molecular mechanism and functional importance of EMT in cancer, there are several mysteries around EMT that remain unresolved. In this Unsolved Mystery, we focus on the variety of EMT types in metastasis, cooperative and collective EMT behaviors, spatiotemporal characterization of EMT, and strategies of therapeutically targeting EMT. We also highlight new technical advances that will facilitate the efforts to elucidate the unsolved mysteries of EMT in metastasis.

## Abstract 2

Epithelial-to-mesenchymal transition (EMT) promotes metastasis. This Unsolved Mystery looks at what is known about different types of EMT, the cooperative behaviors of tumor cells, and spatiotemporal aspects of EMT, and what potential they could hold for developing new therapeutic avenues.

## Introduction

Epithelial-to-mesenchymal transition (EMT) is an embryonic gene program aberrantly activated during cancer progression that facilitates tumor cell detachment from epithelial tissue and subsequent dissemination and metastasis [1]. In 2000, the activation of Snail-mediated EMT in inducing invasion and dissemination of cancer was described for first time [2,3]. These landmark studies began to place a spotlight on the importance of cellular plasticity in cancer metastasis. The study of EMT has blossomed in the following 2 decades, encompassing nearly every aspect of cancer research, with the anticipation that a better understanding of this process may lead to novel therapeutic strategies to prevent or treat cancer metastasis.

EMT has been implicated in metastasis in many different shapes and forms, with significant functional impacts at different temporospatial points of the metastatic process. Initially, and for many years, EMT in cancer was perceived as a simple binary process of transitioning between 2 distinct states, from the initial epithelial polarized state to the mesenchymal-like state, with loss of cell polarity and increased migratory ability. Although this increase in migration and invasion was assumed to promote the metastatic ability of tumor cells, other intriguing metastasis-facilitating properties have also been linked to EMT, such as stem cell properties, immune evasion, metabolic switch, and chemotherapy resistance [1,4]. In 2015, the importance of EMT in metastasis was challenged by studies using specific EMT lineage markers (FSP1 or vimentin) in PyMT-driven or Neu-driven mouse mammary gland tumors, and by genetic knockout of the EMT drivers Snail or Twist in pancreatic cancer mouse models [5,6]. Subsequent studies that used alternative EMT markers or genetic manipulation of other EMT drivers came to opposing conclusions, re-affirming the importance of EMT in metastasis [7,8]. More recently, new computational and cell fate mapping technologies have started to reveal the molecular diversity of the EMT spectrum [9–12], leading to new concepts such as partial EMT, in which cells display markers of both states, or intermediate EMT states, such as hybrid EMT states with a different mix of markers depending on the cancer context [13,14]. The most important conclusion and consensus of these studies was that hybrid EMT states are the most aggressive type of EMT in cancer metastasis. The emergence of single-cell omics and spatial technologies are further uncovering many of these new EMT qualities and types of EMT in much greater detail at unprecedented spatiotemporal dynamic resolution.

The clinical significance of EMT is underscored by recent genomic studies to reconstruct the phylogenetic evolution of metastasis, showing how a significant proportion of metastases are seeded at a very early stage, before clinical manifestation of malignant diseases [15]. This is consistent with earlier experimental evidence showing that EMT can precede tumor formation or occur in very early pre-neoplastic lesions [16–18]. What remains to be seen is whether early disseminated tumor cells in human patients have undergone EMT and whether EMT-targeting therapy in early stage cancers can prevent later occurrence of metastasis.

Altogether, these rapid advances in EMT research have provided a deeper understanding of the complexity of EMT during cancer metastasis, while at the same time raising intriguing new questions about the role of EMT in cancer metastasis and its implications for cancer therapeutics. Solving these mysteries will help improve the prevention and clinical management of metastatic diseases.

### What types and states of EMT drive cancer metastasis?

**Fig 1:**
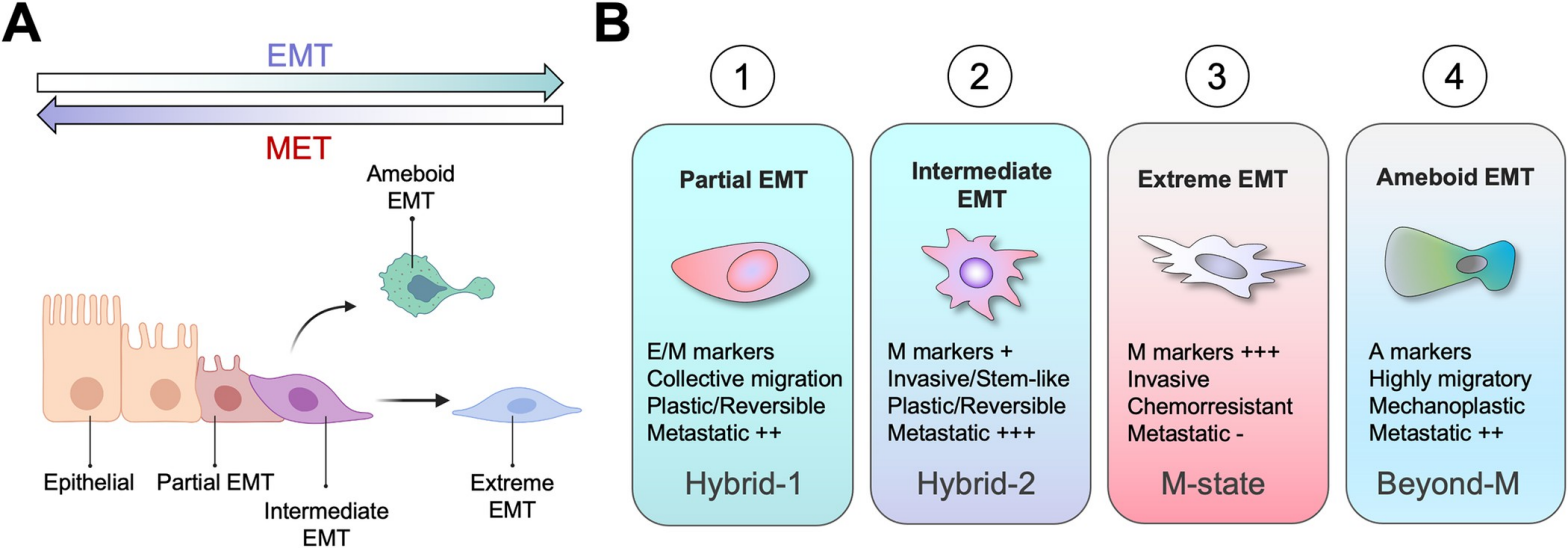
Diverse EMT types are linked to different degrees of plasticity and metastatic potential. (**A**) The spectrum of EMT subtypes with different functional qualities that fall between the classical epithelial (E) and mesenchymal (M) phenotypes, each of these subtypes are defined by the degree of different EMT states through the EMT process. Most of them are easily reversable through MET, except the extreme EMT. (**B**) Classification of EMT into 4 major types with different qualities determining their metastatic potential. (1) Partial EMT involves a hybrid mixture of E and M markers. These are cells in transition between E and M states and have cellular plasticity. This type of EMT is found in collective invasion regions and has metastatic colonization ability. (2) Intermediate EMT refers to a hybrid state that reduces most prominent E markers, gains M markers, but does not reach an extreme M state. It preserves high cellular plasticity, allowing it to revert to MET, and it is endowed with stem cell programs, invasion, and aggressive metastatic ability. (3) Extreme EMT exhibits high expression of classic M markers. This type is highly invasive and chemoresistant but has reduced metastatic abilities due to restricted plasticity in reactivating proliferative states. (4) Ameboid EMT represents a state beyond the M state. These cells exit the EMT transdifferentiation and detour into undifferentiated ameboid-like cells. This state is characterized by high mechanoplasticity, migration, and metastasis ability. Illustration was created in part with Biorender.com.

**Fig 2:**
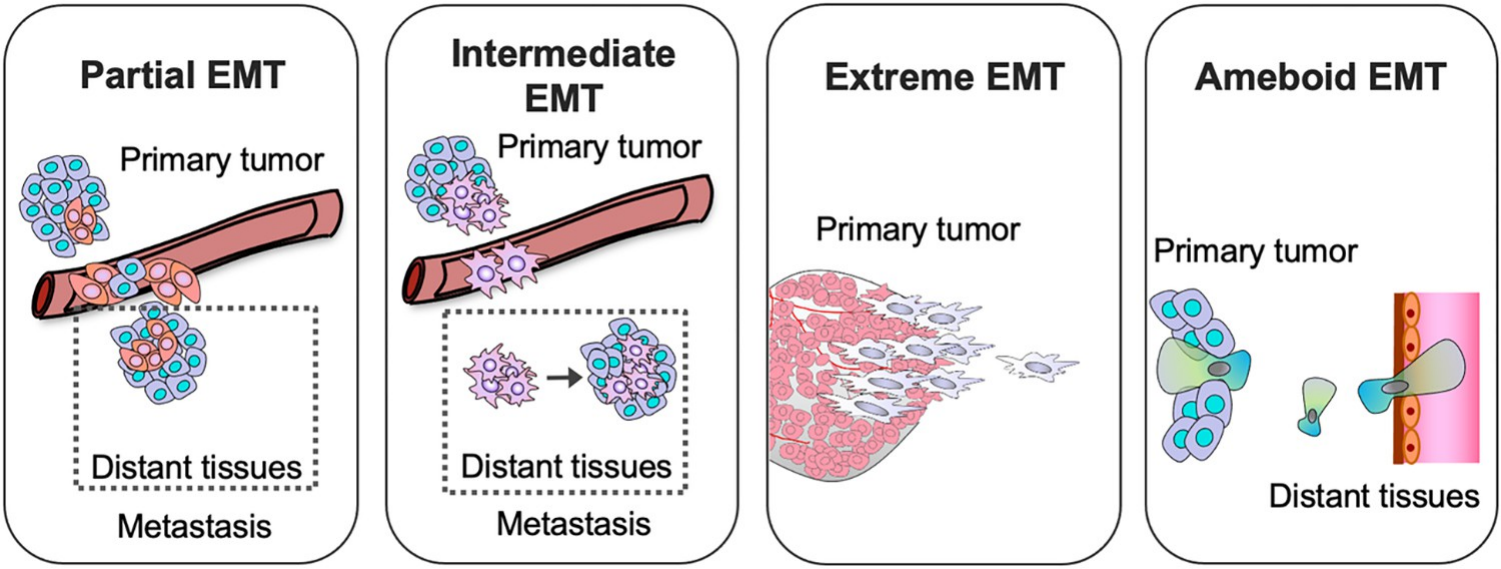
Main implications of the different EMT types in metastasis. (1) Partial EMT is implicated in primary tumor cell dissemination, collective migration, cluster survival in circulation, extravasation, and metastasis colonization. (2) Intermediate EMT has higher plasticity and stemness with higher capacity of tumor initiation in distant metastatic sites. (3) Extreme EMT facilitates tumor cell dissemination in primary tumors, as they are highly invasive. This type of EMT also has high chemoresistance during tumor progression. Its role is less relevant at distant organ sites. (4) Ameboid EMT has particular relevance in primary tumor escape and secondary organ extravasation due to squeezing mobility through reduced tissues spaces.

For many years, research on the role of EMT in cancer has focused on transcription factors driving the loss of epithelial traits, gain of mesenchymal markers, and increased invasion, with the assumption that this was the most important EMT-regulated process that is required for metastasis. However, the importance of EMT in metastasis was not consistently shown in experimental metastasis studies or in analyses using clinical samples from patients with metastatic cancer, and such inconsistency might be due to the diversity of the EMT spectrum and the different criteria used by researchers to characterize EMT. Furthermore, metastasis is a complicated multistep process with many rate-limiting steps beyond invasion; a major bottleneck is the ability to survive the dissemination process and colonize distant tissues in the face of anticancer therapies. As the research on EMT in cancer evolved over time, other malignancy-associated properties beyond tumor invasion, such as stemness [19], immunosuppression [20], and drug resistance [21], have also been associated with EMT. Importantly, during the last decade, several studies using new technologies have shown the existence of many different types of EMT linked to different degrees (intermediate states) of cellular plasticity, with significant implications in metastasis (Figs 1 and 2). While the importance of EMT in metastasis is no longer a topic of major controversy, understanding the exquisite impact of different EMT types and EMT plasticity states on metastasis remains a rapidly evolving area of research.

Much of the confusion and controversies in the study of EMT has stemmed from the simplistic analysis of EMT based on a few molecular markers initially identified from developmental studies, such as using only the loss of epithelial markers E-cadherin or cytokeratin, and gain of mesenchymal markers, such as N-cadherin and vimentin, or expression of EMT driver transcription factors, such as Twist and Snail. These are the canonical markers and detection methods for global EMT changes, but do not capture the complexity of intermediate EMT states. This approach also overlooks the fact that EMT represents a continuum, with cells displaying different degrees and combinations of epithelial and mesenchymal marker expression and the associated spectrum of cellular behavior. In an effort to increase consistency and reproducibility in EMT research, experts in the field have recently reached consensus on how to define and characterize EMT, with a strong emphasis on the characterization of cellular properties with comprehensive molecular analysis, rather than relying on a few readily monitored markers [22]. Specifically, the recommended criteria for EMT definition emphasize the following points: the status of EMT cannot be definitively claimed by using few molecular markers; the primary criteria should be cellular properties in combination with markers; and the EMT functional assessment should be associated with context-specific markers for cancer types and models under study.

New technologies based on single-cell analysis and lineage tracing, together with a better understanding of the EMT process, enables a more refined characterization and definition of the EMT states. For instance, lineage tracing techniques showed that vimentin (a classic mesenchymal marker) is not an accurate marker of EMT-associated metastasis in the MMTV-PyMT breast cancer model. Instead, N-cadherin is a more reliable marker of the EMT phenotype required during metastasis in this model [10]. In other studies, vimentin^+^ EMT resulted in tumor cells that were more invasive and chemoresistant, but not more metastatic [5,23]. Therefore, the validity of a particular EMT marker in indicating metastatic propensity or other malignant features may depend on the specific phenotype under investigation and the cell/model system used in the study. Defining EMT states with associated cellular properties such as increased metastatic potentials will require more comprehensive multi-omics-based integration and characterization of the transcriptomic, epigenomic, and proteomic landscape, along with cellular features such as cell–cell junction, cytoskeletal organization, and cellular polarity.

### Evidence of distinct EMT types

Based on the current knowledge, there are emerging classifications of the EMT types according to their EMT state and metastatic potential (Fig 1A). We classify them here as: partial EMT as a first step of EMT engagement already expressing some mesenchymal markers; intermediate EMT with many mesenchymal features acquired but not yet fully differentiated in a mesenchymal phenotype; extreme EMT as a strong mesenchymal state is acquired; and a divergent branch of EMT acquiring an ameboid state (Fig 1B). The generation of these different EMT types can be a result of several different factors, such as the cell of origin, the dynamics of the process, the temporal moment of the EMT process, the underlying molecular networks, and the epigenetic plasticity to transdifferentiate into “beyond EMT phenotypes” as explained below.

The induction of EMT can be variable, such as spontaneous EMT induction depending on the cell of origin and chromatin states in squamous cell carcinoma (SCC) [24]. In this case, spontaneous EMT is restricted by p63 in interfollicular epidermal cells, while hair follicle cells have more favorable chromatin states to undergo EMT. Pancreatic ductal adenocarcinoma (PDAC) mouse model studies have shown how spontaneous EMT displays 2 subtypes of EMT: the canonical transcriptional suppression of epithelial molecules and an alternative non-transcriptional EMT induction mediated by internalization of epithelial molecules [25]. Moreover, different EMT-driving transcription factors can induce different types of EMT and gene programs despite sharing a common EMT core program [26]. For instance, 2 EMT-driving transcription factor inducers, SNAI1 and PRRX1, lead to different types of EMT, the first with stem cell properties and poor prognosis, and the latter without stem cell properties and an association with a good prognosis in patients [27].

Another dimension of EMT is the spatiotemporal dynamics. Different EMT dynamics generate distinctive EMT qualities. For instance, hysteresis dynamics (when a state is attracted to its original form and displays nonlinear responses to state changing inputs) controlled by a tightly balanced miR-200s–ZEB1 feedback loop leads to the activation of pro-metastatic gene programs, including genes related to stem cell properties and extracellular matrix, that otherwise are not activated by non-hysteresis/linear EMT [28]. While undergoing EMT through hysteresis (nonlinear) or non-hysteresis (linear) dynamics did not show differences in the acquisition of classical EMT markers or their invasive potential, the resulting cell states exhibited different metastatic ability and cellular plasticity [28]. For instance, hysteretic EMT seems to reach an intermediate EMT type with loss of epithelial markers and high cellular plasticity and metastatic potential. These results illustrate how different types and dynamics of EMT can endow tumor cells with different metastatic abilities despite having the same apparent expression of classic EMT markers. Such a diverse spectrum of states therefore cannot be distinguished by only testing a limited number of classic EMT markers. Indeed, single-cell lineage tracing studies of in vivo metastasis have revealed different early and advanced EMT states and markers in an EMT continuum, which are more indicative of aggressive states and metastasis [29].

EMT transdifferentiation beyond canonical EMT-like states and into more differentiated states has also been observed. Intriguingly, long-term EMT induction or EMT in E-cadherin^-^ cells pushed the cells to further transdifferentiate into other specialized differentiated mesenchymal cells, such as adipocytes due to C/EBPα expression [30]. These effects could suppress metastatic abilities, and preclinical proof-of-concept assays indicated this as a possible therapeutic approach. The researchers used MEK inhibitors and antidiabetic drugs to force this EMT conversion into post-mitotic adipocytes to reduce dissemination and lung metastasis in mouse and patient-derived xenograft models. However, these approaches require further careful evaluation, as other studies have demonstrated that MEK inhibitors can lock cells in a hybrid EMT state if TGF-β signaling is not triggered [31].

Another beyond-mesenchymal state (Fig 1) is an extended EMT motile phenotype with amoeboid-like features characterized by high mechanoplastic properties and high individual cell migration. Such a state could facilitate extravasation and metastatic colonization of certain tissue structures [32]. These ameboid-like phenotypes are more metastatic, with enriched stem cell gene programs, and they have been validated in clinical samples as a potential therapeutic target [33,34]. The ROCK–RHO signaling that induces the ameboid phenotypes is also part of the EMT program and has been proposed as an extension of the EMT phenotype [32,35,36]; however, the functional and clinical importance of this phenotype in the context of metastasis still requires further evaluation.

Such great variety in the epithelial–mesenchymal spectrum increases tumor heterogeneity, which is the root cause of treatment failure and resistance. How different cancer cells display distinct EMT types within the tumor microenvironment is still a mystery in most cases. Understanding the cell-intrinsic and microenvironmental regulation of these states will be critical for understanding and predicting the acquisition of the different EMT types in cancer metastasis.

### Epithelial–mesenchymal plasticity and metastasis

EMT is a typical process of cellular plasticity, referring to the reversable ability of dedifferentiation or transdifferentiation of epithelial cells into mesenchymal gene programs with associated morphodynamic and phenotypic changes. In the past few years, epithelial–mesenchymal plasticity has been linked to higher stem cell properties and metastatic ability [11,12,14,37]. This plasticity is in part associated with the ability to revert EMT through mesenchymal-to-epithelial transition (MET), which is an important event in embryonic development, but is also increasingly recognized to be important in cancer metastasis. Once disseminated tumor cells reach distant organs, they revert to a more epithelial-like state, acquiring proliferative potential to facilitate metastatic colonization and outgrowth. Otherwise, non-reversable extreme EMT states would reduce metastatic ability, as has been demonstrated in numerous metastasis studies in animals [38–41] and in more recent lineage tracing studies [11,42].

Intriguingly, studies in embryonic stem cells and normal tissues show how sequential EMT–MET events are necessary for cellular reprogramming [43,44]. In addition, recent studies on cancer cells showed that MET does not revert the cells back to the original epigenetic state, and that the epigenetic memory maintains certain aggressive traits in reverted cells [45]. Sequential reprogramming is aligned with the fact that MET cells can also preserve a stem cell-like state [38,40,46]. By contrast, the lack of plasticity in full/extreme EMT can lead to a differentiated-like state and reduced stem cell properties. This is associated with the loss of metastatic ability observed in cancer cells undergoing full EMT [41] and is a plausible explanation for the lack of pathological evidence of EMT in metastatic tumors. However, the detailed molecular mechanisms by which full EMT, but not hybrid states, reduces stem cell properties are still not known. New single-cell RNA-seq lineage tracing technologies are revealing the existence of different EMT states and their phylogenetic subclonal evolution chronicle during metastasis [29,47]. Full mesenchymal states with less metastatic ability should be further explored to understand the epitranscriptomic network locking them into such terminal EMT states, as this may underlie one of the therapeutic strategies to block the pro-metastatic functions of EMT.

### EMT intermediate states: Definitions and stability

Epithelial–mesenchymal plasticity generates intermediate/hybrid EMT states, which are more plastic, having stem cell-like properties, and more aggressive (Fig 1). However, proper identification and analysis of these states are difficult. In 2018, the Blanpain group identified specific markers of intermediate EMT states in SCC with higher metastatic ability [12]. Of note, 6 different subpopulations or EMT states were found within the EpCAM^-^ cell population, suggesting that within the EMT population, there are many intermediate EMT states. The EpCAM^-^ and CD106^+^CD51^+^ cells were the most plastic and aggressive populations [12]. Other studies have also reported different markers for hybrid/intermediate EMT in different cancer types and models that are associated with higher metastatic ability, such as N-cadherin [10], tenascin-C [11], CD104 and CD44 [48], P-cadherin [49], NFATc [50], NR2F [51], and PDPN and LAMC2 [52]. Whether these are the best markers of hybrid EMT or cellular plasticity, and whether they universally represent EMT plasticity across different cancer types and models requires further investigations.

Recent studies have started to elucidate the molecular mechanisms leading to hybrid EMT phenotypes. For instance, Fat1 deletion stabilizes hybrid EMT in SCC, increasing tumor aggressiveness by activating YAP1 and ZEB1 while preserving epithelial fate [53]. MLL3 loss induces cells to gravitate toward a hybrid EMT state, as it induces EMT in epithelial cells and MET in mesenchymal-like cells through epigenetic H3K27ac marks in IFNγ gene enhancers [54]. ΔNp63/p73 transcriptionally controls hybrid EMT cells, and their loss leads to a less metastatic mesenchymal state in ITGB4^low^ cells [55]. Despite these specific examples, the mechanistic insights into how different cells transition through these different EMT intermediate phases, and how certain states are stabilized or lost, are still open questions under intense research [56]. Therefore, it is important to determine what the epigenetic determinants of such attractor states are to better understand the intermediate states of EMT and have better molecular markers and targets. Another question is whether different cells might display a transitory hybrid state going back and forth in EMT, or just reach steady states. Moreover, the epitranscriptomic networks and self-perpetuating feedback loops that constitute “molecular memory” [57] to maintain these hybrid states are largely unknown and require more research. Advanced spatiotemporal single-cell studies will reveal whether there are multiple or single trajectories for reaching hybrid steady states. Another important effort will be to identify unique features of such hybrid states, such as crucial epigenetic regulators, metabolic state, and cell surface markers that could represent potential targets for therapeutic intervention.

Despite its biological significance, cancer cell plasticity still lacks a standardized definition based on quantitative parameters and scoring systems, which limits mechanistic studies that aim to understand the degree of association between EMT, cell plasticity, and metastasis. How to quantitatively measure EMT plasticity and establish a robust association with the outcomes of metastasis is still an overarching challenge for the field. Therefore, it remains a mystery how relevant cellular plasticity is as a therapeutic opportunity to target EMT in cancer metastasis.

### How does EMT phenotypic heterogeneity affect tumor behavior?

Phenotypic heterogeneity is defined by the presence of different cell populations with different behaviors and functions within tumors. It can be influenced by tumor-intrinsic and tumor-extrinsic mechanisms that dynamically evolve in time and space, and by therapeutic interventions. EMT subtypes and their degrees of plasticity can greatly influence the phenotypic heterogeneity of the tumors by generating distinct cellular behaviors and functions, reflected by the coexistence of distinct EMT types within tumors. In this section, we discuss how EMT phenotypic heterogeneity can lead to aggressive behavior of the whole tumor.

### Implications of EMT in collective and individual cell migration and dissemination

Whether metastases are seeded by cells that disseminated individually or collectively from the primary tumors, and the role of EMT in both scenarios, is still not well determined in patients. Based on experimental models and clinical data studies, both types of dissemination can be responsible for metastasis; however, increasing evidence supports a predominant relevance for collective invasion [58,59]. Intravital imaging studies showed different modes of invasion, including collective invasions, over 20 years ago [60,61]. The collective invasion concept is consistent with the evidence of polyclonal metastasis generated by circulating clusters [62] and the existence of trailblazer phenotypes leading the invasive edge [63].

Recent studies show how partial EMT—defined as displaying a mixture of epithelial and mesenchymal markers (Fig 2)—facilitates collective modes of migration and invasion [25,64]. Partial EMT allows a group of cells to adhere to each other by maintaining some of the intercellular epithelial junctions, while also gaining some of the mesenchymal features, such as back-to-front polarity and actin stress fibers, to facilitate migration. In agreement, lineage tracing studies also report how partial EMT tenascin-C^+^ cells promote collective invasion [11]. These studies are aligned with previous reports describing collective invasion in breast cancer models and patients with different EMT-like traits, including Krt14^+^ and P-cadherin^+^ cells at the invasive tip-fronts [65], which are markers of basal-like states and hybrid EMT. Recently, a multiplex 3D colorectal cancer atlas reported large fibrillar 3D tissue structures with EMT-like features at the tip of the leading edge during invasion [66]. Of note, these large bud-like interconnected structures have been overlooked in cross-sectional 2D histological samples. In addition, topographic single-cell sequencing revealed co-migration of clones into adjacent tissue in invasive ductal carcinoma [67]. Therefore, partial EMT and a continuum in the EMT spectrum may have an important role in collective invasion.

Despite the evidence and functional relevance of collective invasion, individual tumor cells are also found as disseminated circulating tumor cells (CTCs). In addition, individual dissemination is observed in early neoplastic dissemination events [16]. In CTCs from patients with breast cancer, both individual cells and clusters of EMT-like cells are found and associated with a poor outcome [68]. Therefore, tumor cell dissemination may include both individual and collective dissemination alongside the EMT continuum. Of note, individual cells in circulation have a longer lifespan and greater survival than cell clusters, despite the fact that the latter are more efficient in generating metastasis [62]. This observation should be explored further in order to assess the impact of individual disseminated EMT cells compared with clusters in metastasis. Among the different modes of individual EMT-like invasion, the ameboid migration type has important implications in tissue extravasation and colonization of distant organs [32] (Fig 2). Ameboid gene signatures also predict a poor prognosis outcome in breast cancer [69]. Whether ameboid phenotypes are present in CTCs/clusters, or are transitionally induced when tumor cells are entering and squeezing through tissues, are still subjects of further investigation.

Overall, the relevance of EMT within the spatiotemporal combinations of different invasion modes during dissemination requires further preclinical and clinical research to determine which are the most critical for metastasis in different cancer types and organs.

### Cooperative phenotypes in cancer metastasis

Within tumors, due to epigenetic differences or tumor cell location with regards to EMT microenvironmental signals, different cells can undergo EMT with different magnitudes or trajectories. As mentioned above, the EMT continuum generates a multitude of cellular states and phenotypes based on cellular plasticity, which coexist with other types of phenotypic variations to increase intratumoral phenotypic heterogeneity. In fact, lineage tracing studies have shown that intraclonal EMT frequency in different clonal populations in breast cancer contributes to cancer progression [70,71]. Similar to ecological systems, this increase in variability may lead to cooperative behaviors favoring whole tumor capabilities, a phenomenon already observed during tumorigenesis and metastasis [72–74]. Indeed, cooperative effects among different EMT-like cells assuming different roles in the metastatic cascade have been reported previously. In particular, invasive EMT-like cells aid the dissemination [63] of other epithelial tumor cell populations with better tumor-initiating capabilities in distant tissues [38], leading to overt metastasis. Similarly, cooperative cancer cell events were noted in patient-derived xenograft models using barcoding tracing [75]. In addition, cooperative effects in polyclonal metastasis can facilitate immune-evasion during metastasis [76].

The contribution of coexisting and cooperative phenotypes to metastasis is still not well understood. Studying synergistic cell behaviors among tumor cell phenotypes at specific metastatic phases would significantly increase our knowledge of these intercellular dependencies. In fact, whether EMT-like cells are contributing to metastasis individually or by cooperating with other populations is still unknown in clinical settings and could have a strong impact on therapeutic strategies. Experimental models and single-cell lineage tracing technologies should be coupled with clinical validation to provide more insights into these questions.

### EMT induction as a cell population decision-making process

In the embryo, the formation of the parietal endoderm, cell movements during gastrulation, and neural crest formation are the result of a synchronized EMT process emerging in certain cell populations [77] that are extremely well orchestrated in space and time. In gastrulation, it is intriguing that a specific number of cells undergo EMT at the same time and ingress to form the mesoderm in the invagination and epiboly spreading movements. However, in mice, recent studies have shown asynchronous ingression during gastrulation due to physical constriction at the primitive streak [78]. Nonetheless, we can find the origin in the “organizer” groups of cells, such as the Nieuwkoop center and Spemann organizer, which demarcate the inception of EMT in *Xenopus* [79] by releasing Wnt/β-catenin and Nodal/TGF-β signals. These signals can dictate the spatiotemporal coordination of EMT during gastrulation [79,80].

Similar structures have not been identified in cancer, and it is still unknown whether they exist during EMT in cancer. EMT is not specifically restricted to a particular moment or space within tumor development, instead it seems to be spontaneously driven by external stimuli at the invasive edge of tumors and regions with extracellular matrix contact. Studies using intravital microscopy have demonstrated the occurrence of spontaneous EMT of individual cells disseminating from MMTV-PyMT breast tumors [81,82], and also how TGF-β can coordinate the local switch to EMT-like single-cell motility [83]. A better understanding of collective decisions relies on the identification of cell population sensor systems. Interestingly, E-cadherin—the loss of which is a hallmark of EMT—works as a sensor molecule for cell population density and modulates responses to growth factors in the entire population [84–86]. In addition, computational simulations of cell population behavior have predicted that the spreading of EMT and signal-sensing from neighboring cells are modulated by the miR-200–ZEB1 feedback loop. This neighborhood spreading effect was experimentally demonstrated in cultured cells in vitro [28]. Overall, these studies suggest that tumor cells might coordinate EMT in a group fashion in cancer.

Synchronized populations require checkpoint mechanisms and feedback loop controllers working at the population level, and thus functioning as a higher order entity or complex system. An open complex system (when a system as a whole is more than the sum of its parts) is composed of many agents of the same type, such as cells, which act stochastically and individually in a self-serving way yet together lead to emergent behaviors. Bacterial “quorum-sensing,” which senses bacterial cell density, exemplifies a social response based on chemical communication in the population, which is transduced to gene expression changes in cells [87,88]. In metastasis, quorum-sensing-like behavior has been suggested to lead to ovarian cancer metastatic growth [89]. Collective behaviors, such as “swarm-like behavior” or quorum sensing, and the relevant decision-making processes remain mostly speculative in EMT and cancer metastasis research, and one of the most challenging mysteries of EMT is linking evolutive behaviors with metastasis. However, multidisciplinary collaborations involving new technologies in single-cell lineage tracing, quantitative imaging, and mathematical deep learning modeling should be able to reveal and answer some of these questions in future years.

### How to target EMT in cancer therapeutics?

The involvement of EMT in cancer metastasis was initially questioned, as metastatic lesions often maintained mostly epithelial features. This doubt has been addressed to some extent with the observation that EMT is a temporary and reversible progress that is often observed at the invasive front of the tumor [90], during circulation [68,91], or in initial seeding at distant sites [18]. With increasing recognition of the critical roles of EMT and MET in cancer, there has been growing interest in using EMT in prognostic/predictive markers or as a therapeutic target in clinical settings.

One application of EMT and MET markers is as prognostic indicators in various cancer types. Monitoring these transitions in patient samples can aid in predicting disease progression and determining appropriate treatment strategies. For example, in breast cancer, the presence of EMT markers such as vimentin and N-cadherin, as well as drivers of EMT, such as Snail and Twist, is associated with an aggressive phenotype and poor clinical outcomes [92]. Application of EMT gene signatures, rather than single markers, provides a more comprehensive evaluation of EMT status in clinical samples. Thiery and colleagues developed a genomic EMT scoring method and used it to show correlations between EMT and poorer disease-free survival in ovarian and colorectal, but not breast, carcinomas [93]. Differential responses between epithelial-like and mesenchymal-like ovarian cancers to therapeutic regimes administered with or without paclitaxel in vivo was also observed. Hybrid EMT scores have also been associated with worse survival outcomes [94]. Similarly, pan-cancer EMT signature analysis in clinical tumor samples across different cancer types revealed a strong correlation between EMT and immune checkpoint activation [95,96]. Using EMT signature analysis in patients with non-small cell lung carcinoma in clinical trials and in animal model studies, EMT-like mesenchymal cells showed significantly greater resistance to EGFR and PI3K/AKT pathway inhibitors, independent of EGFR mutation status, but more sensitivity to certain chemotherapies [97]. Mesenchymal cells also expressed increased levels of the receptor tyrosine kinase AXL and greater sensitivity to the AXL inhibitor SGI-7079. The EMT signature also predicted 8-week disease control in patients with non-small cell lung carcinoma receiving erlotinib but not other therapies. These promising studies suggest possible applications of such analysis for guiding the application of chemotherapy, immunotherapy, and targeted therapy.

**Fig 3:**
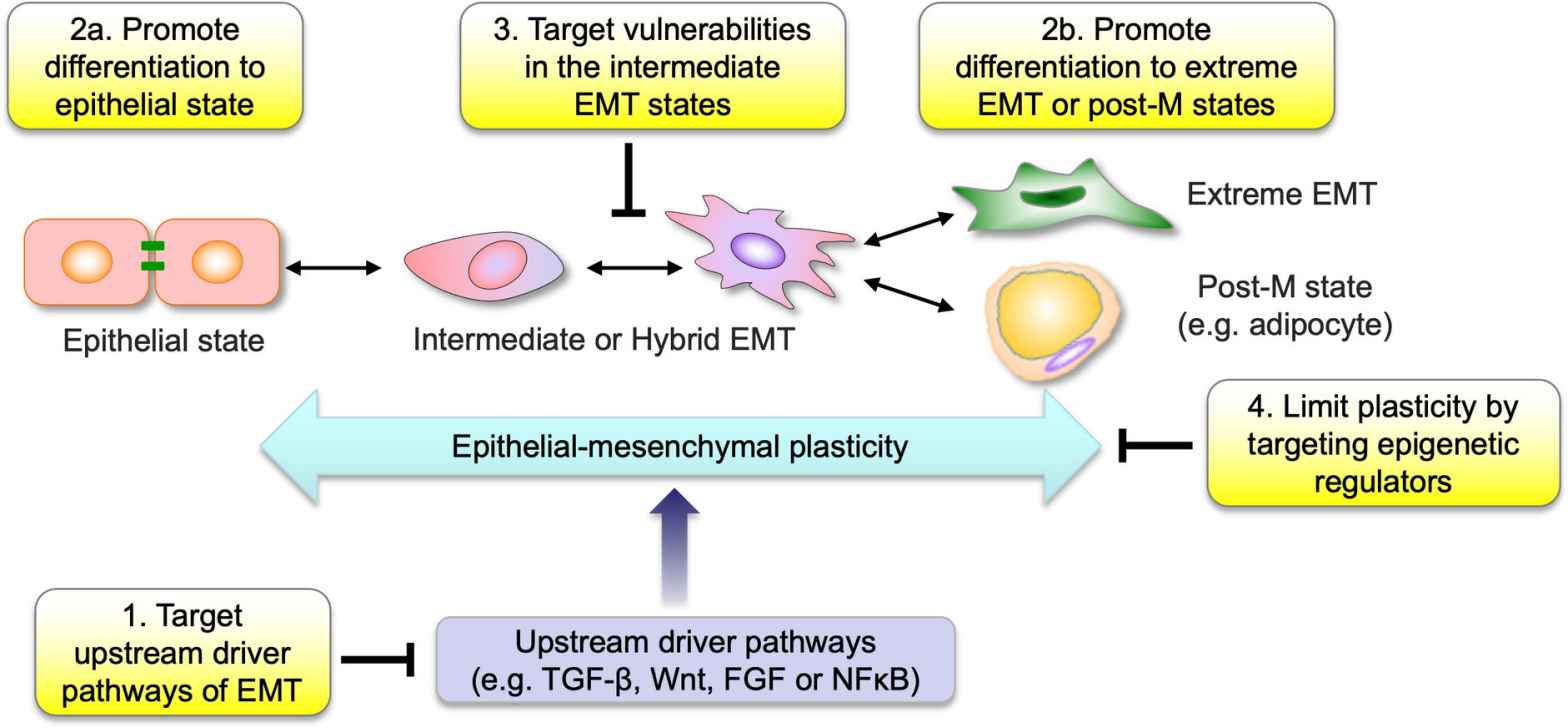
Strategies to target EMT and cellular plasticity in metastasis. Many oncogenic drugs are currently under development that target EMT upstream pathways, such as TGF-β, Wnt, FGF, or NFκB, although it will be challenging to discern how much of the therapeutic benefit is due to inhibition of EMT. An alternative strategy is to drive cells in highly metastatic hybrid or intermediate EMT states into differentiating into an epithelial state or into a terminally mesenchymal or post-mesenchymal state, such as adipocytes. The third approach identifies vulnerabilities of cells at the intermediate or hybrid EMT states as potential therapeutic opportunities, either as single targets or in combination with other standard therapies. The fourth strategy is to limit cellular plasticity and inter-conversion between intermediate EMT states by targeting epigenetic regulators or molecular memory circuits that confer such plasticity to tumor cells.

There are several avenues to targeting EMT in cancer therapeutics: targeting EMT drivers and upstream pathways; limiting cellular plasticity and acquisition of intermediate EMT states; targeting vulnerabilities of cells in the intermediate or hybrid EMT states; and pushing cells to an extreme EMT state or terminally mesenchymal or post-mesenchymal state (Fig 3). Promising therapeutic targets associated with EMT and cellular plasticity include key signaling pathways such as the TGF-β pathway, the Wnt signaling pathway, and the Notch pathway. Small molecule inhibitors and monoclonal antibodies targeting these pathways are being developed and tested in preclinical and clinical settings. For instance, targeting TGF-β, the key EMT-driving pathway, has shown significant therapeutic benefits [98]. In pancreatic cancer, genetic depletion of the EMT transcription factor ZEB1 suppressed cancer stemness, metastatic colonization capacity, and phenotypic/metabolic plasticity of tumor cells [7]. Targeting vimentin with a linear peptide, aptamers or natural products has also shown promising results in preclinical models [99]. Recently, netrin-1 was found to be up-regulated during EMT, and the humanized anti-netrin-1 antibody NP137 can block metastatic progression in SCC and endometrial cancer mouse models [100,101]. Importantly, NP137 is already in clinical trials for advanced, refractory solid tumors (NCT02977195), with early analysis showing a shift of tumors toward a more epithelial phenotype [102], consistent with mouse model observations. These examples highlight the potential of EMT-targeted therapies in preventing cancer progression and improving treatment outcomes.

Exploiting cellular plasticity is another avenue for therapeutic targeting. Cancer cells with high plasticity can switch between epithelial and mesenchymal states, making them adaptable to different microenvironments and resistant to treatment. In breast cancer, therapies targeting CSCs through EMT inhibition have shown promise in reducing tumor growth and preventing metastasis in mouse models. For example, salinomycin, a potassium ionophore antibiotic, was identified to have selective toxicity and induce increased epithelial differentiation in the EMT-like breast cancer stem cell subpopulations in preclinical studies [103]. Such an effect of salinomycin might be due to its ability to promote the degradation of the Wnt coreceptor LRP6 and subsequent inhibition of Wnt signaling [104]. Instead of reverting EMT and differentiating CSCs to an epithelial state, another approach of differentiation therapy is to push the cells into a post-mitotic and terminal mesenchymal state, such as transdifferentiation of EMT-derived breast cancer cells into adipocytes through a combination therapy with MEK inhibitors and the anti-diabetic drug rosiglitazone. This combination therapy has been shown to inhibit primary tumor invasion and metastasis formation in various preclinical models [30].

Combination therapies that target cells in EMT states have also gained attention. This approach aims to induce MET in cancer cells, rendering them more susceptible to chemotherapy, immunotherapy, and targeted therapy. For example, the TGF-β inhibitor SB-431542 or antibodies against TGF-β receptors increase sensitivity to carboplatin [105]. Such combinatorial strategies hold potential in overcoming treatment resistance and improving patient outcomes; however, they should be carefully assessed, as MET facilitates metastatic potential at distant sites.

While significant progress has been made in targeting EMT in cancer, several challenges remain. One challenge lies in the heterogeneity and plasticity of tumor cells, as they can dynamically switch between epithelial and mesenchymal states, making it difficult to effectively target a constantly evolving population of cancer cells. Additionally, the timing and context-specificity of EMT during tumor progression pose challenges for therapeutic intervention. EMT can occur at different stages of cancer development and in response to various microenvironmental cues, and may play opposite roles in different steps of metastatic progression. Therefore, targeting EMT requires a nuanced understanding of when and how to intervene, and whether it could be applied in the clinical practice. Moreover, the complexity of EMT signaling networks and crosstalk with other pathways further complicates therapeutic targeting and evaluation of the exact therapeutic benefit of targeting EMT. The development of new therapeutic strategies that target key nodes in the phenotypic plasticity network will be critical for new therapeutic strategies in patients with metastases. Lastly, the lack of reliable biomarkers for EMT and MET poses a challenge in patient selection and monitoring treatment response. Overcoming these challenges will require interdisciplinary efforts, advanced technologies, and further insights into the intricacies of EMT regulation and its implications in cancer biology.

### Conclusions and future directions

As an important non-mutational way for tumor cells to gain mobility, cope with various stresses during the metastatic cascade, and adapt to new environments, epithelial-to-mesenchymal plasticity holds a key role in metastasis. Over the past decade, the concept of EMT in metastasis has rapidly evolved, expanding to new forms, functions, and consequences of EMT that strongly affect tumor aggressiveness and metastasis. The emergence of single-cell technologies and the application of mathematical modeling have revealed multiple trajectories for acquiring a diverse range of EMT states and have thus forced the scientific community to revisit the concept of EMT in metastasis. The old concept of EMT as a binary switch has now been replaced with the consensus that EMT represents a spectrum of cell states displaying different degrees and combinations of epithelial and mesenchymal features. Moving forward, challenges remain regarding how to define, measure, and catalog EMT states and their stability, and how to link accurate quantitative definitions of each intermediate state with cellular characteristics, such as stemness, invasiveness, immunogenicity, metastatic potential, and vulnerability to various therapeutic agents. Furthermore, understanding which molecular events lead to a locked EMT state will help us to understand the loss of plasticity and possible opportunities for therapeutic intervention.

It is now clear that EMT and MET are highly dynamic and temporal process that occur in specific spatiotemporal windows, and that end-point analysis rarely captures critical events with the most significant functional impact on the final outcome of metastatic diseases. New single-cell and lineage tracing technologies have provided improved resolution of EMT, enabling the dissection of EMT into types and states. A more thorough histological exploration using spatial technologies and the development of tools to analyze and record cellular plasticity in living tissues are critical for future advancements in this field. The application of machine learning and artificial intelligence to analyzing and interpreting the wealth of information generated by such new technologies will likely reveal key cellular states and their regulatory networks that could be most effective targets for therapeutic intervention.

In cancer research, success in therapeutic development is the ultimate validation of the mechanistic understanding of cancer. In this regard, the study of EMT still faces an uphill battle to achieve this lofty goal. The diversity of the EMT spectrum and the heterogeneity of tumor cells displaying different states along the spectrum in the same patient will likely undermine the development of EMT-targeting therapeutics. Instead of striving to identify and target a key driver of EMT for cellular plasticity that holds a universal role in diverse cancer types, efforts may need to be directed toward a specific disease in which an EMT regulator has been functionally validated and consistently implicated in patient cohorts that can be identified with specific tumor markers. Clinical trials to test such therapeutics will need to have clearly defined end-points based on biological mechanisms, with easily accessible markers to evaluate on-target effects and pharmacodynamics. As EMT is also involved in many physiological processes, careful evaluation of toxicity and the correct therapeutic window will also be key for success in drug development.

In summary, we are now facing an exploration of the EMT multiverse that encompasses a diverse range of states, trajectories, dynamics, forms, and functions, raising new questions of increasing complexity. The consensus on how to define EMT as a spectrum of cellular states, alongside the application of new spatial and single-cell technologies, is a cornerstone for continuing progress in solving the remaining unsolved mysteries of EMT research and translating new insights into better treatments for cancer.

## Abbreviations

CTC: circulating tumor cell
EMT: epithelial-to-mesenchymal transition
MET: mesenchymal-to-epithelial transition
PDAC: pancreatic ductal adenocarcinoma
SCC: squamous cell carcinoma
